# Cognitive Processes of Probabilistic Prediction in Reading: Language Model Surprisal Across Model Sizes, Token Granularity, and Reading Paradigms

**DOI:** 10.3390/jintelligence14080171

**Published:** 2026-08-01

**Authors:** Shuting Liu, Yong Mei

**Affiliations:** School of Foreign Languages, Hubei University, No. 368 Youyi Avenue, Wuchang District, Wuhan 430062, China; 202421103010607@stu.hubu.edu.cn

**Keywords:** surprisal, cognitive processes of reading, large language models, GPT 2, Llama, eye tracking, reading assessment, Natural Stories Corpus, Provo Corpus

## Abstract

Surprisal, the negative log probability of a word given its context, is the dominant computational metric for quantifying reading difficulty and a common item difficulty estimator in reading research. Yet how the language model family, surprisal granularity, and corpus type jointly shape the surprisal–reading time link remains unclear. We conducted a secondary analysis of two public English reading corpora: the Natural Stories Corpus (self-paced reading, 181 readers) and the Provo Corpus (eye tracking with cloze norms). Surprisal was computed from GPT 2 (Small, XL), Llama 2 (7B, 13B), and Llama 3 (8B), together with a 5-gram baseline and human cloze norms. Linear mixed-effects models tested the baseline contribution of neural surprisal, inverse scaling within model families, word-level versus sub-word aggregation, and the linking function shape. Neural surprisal contributed reliable variance above a strong baseline in both corpora. A clear inverse scaling pattern emerged: GPT 2 Small produced the largest fit improvements, exceeding GPT 2 XL, Llama 2 13B, and Llama 3 8B. Word-level aggregation outperformed sub-word aggregation, especially for measures of early lexical access. Non-parametric analyses supported an approximately linear linking function, and cloze norms carried information not fully captured by neural surprisal. These findings show that larger models are not automatically better cognitive models of reading and that surprisal granularity is not a neutral analytic choice.

## 1. Introduction

Reading is the cognitive pillar of formal education, and moment-to-moment variation in how long a reader’s eye lingers on each word is one of the most informative behavioural signatures of the underlying language processing system (Shain, 2024; Staub, 2015). A central empirical generalisation that has emerged from decades of psycholinguistic research is that less predictable words are read more slowly than more predictable ones (Brothers & Kuperberg, 2021; Shain et al., 2024; Wilcox et al., 2023). The information-theoretic formalisation of this generalisation, known as surprisal theory, posits that the processing cost incurred by a word in context is proportional to the negative log probability of that word given its preceding context (Hale, 2001; Levy, 2008). Surprisal theory furnishes a quantitative bridge between probabilistic models of language and the cognitive processes of reading, and it has become the de facto interface through which modern language models contribute to the study of human sentence processing (Shain et al., 2024; Smith & Levy, 2013; Wilcox et al., 2023).

The rapid maturation of large pre-trained autoregressive language models, ranging from the GPT 2 family (Radford et al., 2019) to the Llama 2 and Llama 3 families, has profoundly enlarged the toolkit available for estimating contextual word probabilities. A natural expectation, voiced both inside and outside the cognitive sciences, is that larger and more capable language models, which assign higher probabilities to held-out text, should also provide more human-like estimates of moment-to-moment processing difficulty (Goodkind & Bicknell, 2018; Wilcox et al., 2020). Recent work has called this expectation into question. A series of studies has now shown that, within architecturally homogeneous families of pre-trained Transformer models, the larger and lower-perplexity variants yield surprisal estimates that are systematically less predictive of human reading times  (Boeve & Bogaerts, 2025; Oh et al., 2022; Oh & Schuler, 2023, 2025; Shain et al., 2024). This inverse scaling finding sits in tension with the older positive relationship between language model quality and psychometric fit reported for smaller models (Goodkind & Bicknell, 2018; Hao et al., 2020), and it has been replicated across languages, model families, and reading regimes (Boeve & Bogaerts, 2025; Liu et al., 2024; Shubi et al., 2024; Wilcox et al., 2023). Whether the inverse scaling phenomenon is robust to data leakage (Oh et al., 2025) or merely an artefact of specific training corpora is now an active subject of debate.

A second, methodologically subtler question concerns the granularity at which surprisal should be computed. Modern Transformer-based language models do not tokenise text at the level of whole orthographic words; they operate over sub-word units whose boundaries do not coincide with the boundaries presented to a human reader. In consequence, a research design that maps sub-word surprisal to the next fixation duration reflects an underlying and potentially distorting alignment decision. Recent evidence suggests that aggregating sub-word surprisal up to the whole word level changes the strength and even the direction of fit to both naturalistic reading times and to structurally challenging constructions, such as garden paths (Liu et al., 2024; Nair & Resnik, 2023; Oh & Schuler, 2025). The psychometric consequences of token granularity are particularly acute in eye tracking, where early lexical access measures are word-indexed by construction.

A third underexposed dimension is the dual role of the corpus type. The Natural Stories Corpus (Futrell et al., 2021) provides self-paced reading times for ten English passages edited to overrepresent rare syntactic constructions, and it has become a standard benchmark for testing surprisal theory at the boundaries of grammatical expectation (Oh & Schuler, 2023; Oh et al., 2025; Shain et al., 2024). The Provo Corpus (Luke & Christianson, 2018) provides eye tracking records over short paragraphs together with detailed human cloze probability norms, and it is uniquely suited to comparing model-based surprisal against the human reference signal that surprisal is intended to approximate (de Varda et al., 2024; Shain, 2024). To date, comparatively few studies have systematically contrasted model fits across both corpora using a unified analytic pipeline, even though doing so would allow the careful separation of the effects of the reading paradigm (self-paced vs. eye tracking), text genre (syntactic stress test vs. naturalistic prose), and reference signal (model surprisal vs. cloze) on the surprisal–reading time link.

The present study addresses these three dimensions in a single, integrated analysis. We perform a fully secondary analysis of two publicly available reading corpora, the Natural Stories Corpus and the Provo Corpus, and we evaluate eleven sources of word-level predictability. The sources span (i) a 5-gram statistical baseline, (ii) human cloze predictability norms from Provo, (iii) four variants of GPT 2 differing in parameter count, (iv) Llama 2 7B and Llama 2 13B, and (v) Llama 3 8B. We additionally contrast sub-word and whole-word surprisal aggregations and ask whether the choice of aggregation modifies the psychometric leaderboard. By holding the statistical pipeline constant across corpora and across model families, we provide a clean empirical map of how the surprisal–reading time link varies as a function of the model family, model size, granularity, and reading paradigm.

The individual empirical components of our investigation, including the inverse scaling phenomenon, the granularity contrast, and the role of cloze norms, have each received attention in prior work. The integrative contribution of the present study lies in subjecting these components to a single, jointly controlled analytic pipeline that holds the statistical methodology constant across two complementary corpora, eight neural estimators spanning three model families and four orders of magnitude in parameter count, and multiple dependent measures from distinct reading paradigms. This unified design allows us to assess whether the inverse scaling pattern generalises beyond the self-paced reading data on which it was originally documented, whether token granularity interacts with model size and with reading measure sensitivity, and whether cloze norms retain unique explanatory value when evaluated against the same baseline as neural surprisal. No prior study has addressed these questions simultaneously under matched analytic conditions. In addition, we extend the empirical record by reporting temperature scaling analyses, effect sizes in terms of marginal $R^{2}$ changes, cloze-by-surprisal interaction tests, and expanded cross-validation and generalised additive analyses across all estimators, none of which have been reported in this combination previously.

Beyond methodological integration, the study makes three substantive contributions to the theory of probabilistic prediction in reading. The inverse scaling analyses, supplemented by temperature scaling and word type decomposition, provide evidence that the discrepancy between model quality and psychometric fit reflects a combination of distributional overconfidence and word-specific memorisation, advancing the theoretical understanding of why surprisal from larger models departs from human processing costs. The granularity analyses provide evidence that the cognitive unit over which probabilistic prediction operates during reading corresponds to the whole orthographic word rather than to the arbitrary sub-word tokens used by contemporary language models—a conclusion with direct implications for theories of lexical access during reading. The cloze analyses demonstrate that human contextual expectations and neural model predictions capture complementary rather than competing dimensions of predictability, indicating that current language models, despite their superior perplexity, do not yet reflect the full space of information that human readers bring to bear on prediction.

Our motivation aligns with the broader interest in the cognitive processes that underlie language and reading. A clear methodological understanding of which language models best track moment-to-moment processing difficulty in skilled adult readers is a necessary preliminary step before the same instruments can be responsibly adopted in any applied reading research context (Liu et al., 2024; Oh & Schuler, 2023). The granularity question that we raise has practical implications for reading research that uses surprisal as an item difficulty estimator. The joint corpus design clarifies whether the inverse scaling phenomenon generalises beyond the self-paced reading paradigm and whether the cloze signal that has long anchored reading research (de Varda et al., 2024; Luke & Christianson, 2018) remains a competitive predictor in the era of large language models.

The remainder of the paper is organised as follows. Section 2 reviews surprisal theory, the inverse scaling phenomenon, the token granularity question, and the role of cloze norms and then derives four research questions and four aligned exploratory predictions. Section 3 describes the corpora, the language models, the surprisal extraction pipeline, the control variables, and the linear mixed-effects analytic strategy. Section 4 presents corpus descriptive statistics, baseline analyses, neural surprisal contributions, the within-family scaling analyses, the granularity analyses, and a non-parametric linking function analysis. Section 5 integrates the findings into theoretical and methodological implications for the cognitive processes of reading. Section 6 outlines limitations and future directions. Section 7 concludes.

## 2. Theoretical Background and Literature Review

### 2.1. Surprisal Theory and the Cognitive Processes of Reading

Surprisal theory (Hale, 2001; Levy, 2008) casts the difficulty of processing a word $w_{t}$ in context $C_{t}$ as proportional to its information content,

(1) $$\mathrm{surprisal}\left(w_{t}|C_{t}\right)=-\log P\left(w_{t}|C_{t}\right),$$

under a probabilistic model of the language. The theoretical appeal of this formulation lies in three properties. Surprisal is grounded in resource allocation reasoning: a comprehender who maintains a probability distribution over upcoming material must redistribute mass as new evidence arrives, and the cost of this redistribution is the Kullback–Leibler divergence between the prior and posterior, which reduces to surprisal under standard assumptions (Levy, 2008).

In addition, surprisal links naturally to information-theoretic measures of predictability that can be estimated from any probabilistic model of language, from n gram models through probabilistic grammars to modern neural language models (Hao et al., 2020; Michaelov et al., 2024; Wilcox et al., 2020). Surprisal also makes a quantitative prediction about the word-by-word reading time, which has been extensively tested against self-paced reading and eye tracking corpora (Shain et al., 2024; Smith & Levy, 2013; Wilcox et al., 2023).

Two further generalisations of surprisal theory have received considerable empirical support. The better-known example is the logarithmic—equivalently linear in surprisal—linking hypothesis: across a wide range of reading datasets, the processing time grows approximately as a linear function of surprisal, rather than superlinearly or as a step function (Shain et al., 2024; Smith & Levy, 2013; Wilcox et al., 2023). Recent work, however, has challenged this consensus: Brothers and Kuperberg (2021) present evidence from controlled self-paced reading and cross-modal picture naming for a linear—rather than logarithmic—relationship between the cloze probability and reading time. The reconciliation of these views remains an active topic, with reanalyses of the same datasets using neural language model surprisal recovering a linear surprisal effect at the upper end of the predictability range (Shain et al., 2024). The second generalisation is the contextual entropy hypothesis: in addition to the surprisal of the actually observed word, the expected surprisal of upcoming material, equivalent to the contextual entropy of the next word distribution, predicts the incremental processing time (Pimentel et al., 2023; Wilcox et al., 2023).

### 2.2. The Inverse Scaling Phenomenon

For most of the first two decades after Hale’s original proposal, empirical work supported a positive relationship between language model quality and the psychometric value of the surprisal that it yields (Goodkind & Bicknell, 2018; Hao et al., 2020; Wilcox et al., 2020). Goodkind and Bicknell (2018) formalised this as the conjecture that surprisal’s predictive power for reading time is a tight linear function of language model perplexity. With the arrival of large pre-trained Transformer language models, however, the picture changed. Oh and colleagues (Oh et al., 2022; Oh & Schuler, 2023), examining GPT 2, GPT Neo, and OPT variants, reported that the larger and lower-perplexity variants produced surprisal estimates that were systematically less predictive of self-paced reading times on the Natural Stories Corpus and of eye tracking go-past durations on the Dundee Corpus. Subsequent work has replicated this inverse scaling phenomenon across a broader set of model families, training data sizes, and reading datasets (Boeve & Bogaerts, 2025; Liu et al., 2024; Oh & Schuler, 2025; Oh et al., 2025; Shain et al., 2024).

A memorisation account proposes that larger models assign disproportionately low surprisal to rare training material, including named entities and idiosyncratic collocations, which human readers nonetheless process slowly (Oh & Schuler, 2023). This theory predicts that the inverse scaling effect should be more pronounced on low-frequency content words than on high-frequency function words and more pronounced on words that are likely to appear verbatim in web-scale training corpora.

A calibration account emphasises that larger models become overconfident relative to the human reader: their probability distributions are sharper than those of an ideal observer, and temperature scaling that softens the output distribution recovers part, although not all, of the lost fit (Liu et al., 2024). The calibration account makes the testable prediction that applying an appropriately tuned temperature parameter should narrow the gap between smaller and larger models and that the residual gap after calibration reflects factors beyond confidence miscalibration. Particle filter theories argue that human comprehenders maintain only a small number of structural hypotheses in parallel, which produces non-linearities and amplifications in difficulty that smoothly probabilistic surprisal cannot capture. Alternative algorithmic theories such as sampling-based incremental processing predict superlinear linking functions (Hoover et al., 2023), providing a principled account for some of the discrepancies. The inverse scaling phenomenon has practical consequences: it implies that, for psycholinguistic research, the default reflex of selecting the largest available model is counterproductive.

Despite the growing body of evidence, no prior study has jointly tested the memorisation, calibration, and granularity aspects against reading data from both self-paced and eye tracking paradigms. Disentangling these mechanisms requires not only within-family scaling comparisons but also targeted analyses of word types and temperature-adjusted surprisal estimates, both of which we incorporate into the present design.

### 2.3. Token Granularity and Word-Level Aggregation

Modern Transformer language models do not operate over whole words. Their tokenisers, which include byte pair encoding and Unigram Language Model variants, segment words into sub-word units chosen to balance the vocabulary size and out-of-vocabulary coverage. As a consequence, a phrase such as “the unbelievable consequences” may be tokenised into five or six sub-word pieces, several of which carry surprisal values that do not correspond to any cognitively transparent unit. When such sub-word surprisals are used to predict reading times measured at the level of whole words, the analyst must make an implicit alignment decision. Two common choices are either to assign the surprisal of the first sub-word to the entire word or to sum the sub-word surprisals to obtain a word-level surprisal. The latter has a clean information-theoretic interpretation: by the chain rule of probability, the sum of sub-word surprisals for the pieces of a word equals the surprisal of the whole word given its left context.

Recent work has shown that the alignment choice is not psychometrically neutral. Aggregating sub-word surprisals to the whole-word level yields a more linear linking function and a more sensible response to garden path constructions, where coarser tokenisations track syntactic disambiguation more faithfully (Nair & Resnik, 2023; Oh & Schuler, 2025). Importantly, the temperature scaling effect documented by Liu et al. (2024) is largely driven by multi-sub-word words, suggesting that calibration interacts with granularity in non-trivial ways. The granularity question has not yet been systematically examined against eye tracking measures that are particularly sensitive to early lexical access, such as the first fixation duration and gaze duration on the target word, in which the early portion of the word’s surprisal may matter disproportionately. We therefore include a granularity contrast as a core empirical question rather than as a robustness check.

A natural extension of the granularity question is whether aggregation at the morpheme level, rather than at the whole-word level, might offer a psycholinguistically more principled unit of analysis, given that morphological boundaries are cognitively transparent and influence lexical access (Nair & Resnik, 2023). For English, which has a relatively sparse inflectional morphology, the distinction between morpheme-level and word-level aggregation is less consequential than it would be in morphologically rich languages such as Turkish or Finnish, where a single orthographic word may contain several productive morphemes. We return to morpheme-level aggregation as an explicit priority for future research in Section 6, and we focus the present analysis on the contrast between whole-word summation and first-sub-word assignment, which are the two choices most commonly adopted in the existing literature (Liu et al., 2024; Nair & Resnik, 2023; Oh & Schuler, 2025).

### 2.4. Naturalistic Corpora and Cloze Norms

Two complementary publicly available English reading corpora are particularly suited to the present investigation. The Natural Stories Corpus (Futrell et al., 2021) comprises ten passages totaling 10,245 word tokens, edited to oversample rare syntactic constructions while remaining fluent, together with self-paced reading times from 181 adult readers. The corpus is the primary benchmark in contemporary surprisal research (Boyce & Levy, 2023; Oh & Schuler, 2023; Oh et al., 2025; Shain et al., 2024) and provides a controlled stress test of model predictions in the regions of grammatical space where competing theories most clearly diverge. The Provo Corpus (Luke & Christianson, 2018) comprises 55 short paragraphs containing 2689 word tokens read by 84 participants while their eye movements were recorded; crucially, the corpus is accompanied by cloze predictability norms collected from a separate norming sample, including the predictability of the target word’s full orthographic form and of the word’s morphosyntactic and semantic components.

The pairing of self-paced reading data with eye tracking data, and of model-derived surprisal with human cloze norms, allows a clean test of two questions that are of substantive interest in the cognitive processes of reading.

One question is whether the model-based surprisal advantages observed on self-paced reading times persist when the dependent variable is an eye tracking measure that more directly indexes moment-to-moment cognitive processes.

A complementary question is whether the human cloze norm continues to compete favourably with state-of-the-art neural surprisal as a predictor of eye movement given that a cloze provides a noisy but behaviourally grounded estimate of human contextual expectation (de Varda et al., 2024; Luke & Christianson, 2018; Shain, 2024).

An important caveat is that cloze norms and model surprisal are not directly comparable in terms of measurement error or construct validity. Cloze norms are collected offline through a continuation task that elicits metalinguistic judgments rather than online processing, and they are subject to sampling noise, strategic guessing, and ceiling or floor effects at extreme predictabilities. Model surprisal, by contrast, is a deterministic function of the model and the input text. Interpreting the residual contribution of clozes over model surprisal therefore requires distinguishing between genuine differences in the constructs captured and differences attributable to measurement properties. We address this issue empirically by reporting the correlation between cloze and model surprisal estimates and by testing their interaction, both of which help to contextualise the additive log-likelihood results.

### 2.5. Methodological Relevance to Broader Reading Research

Beyond its role as a theoretical benchmark, the choice of surprisal estimator has downstream methodological consequences for any reading research that uses predictability as a covariate or item difficulty metric. Eye movement signatures of probabilistic prediction have been shown to vary across populations and across the lifespan (Pereira et al., 2024; Schuckart et al., 2026), and broader reviews underscore that the functional consequences of predictability remain an active area of theoretical debate (Wong et al., 2025). Crucially, the instrument used to quantify predictability propagates directly to the conclusions drawn from such analyses: a poorly calibrated surprisal estimator can mistake an artefact of the estimator for a feature of the data under investigation (Liu et al., 2024; Oh & Schuler, 2023). Establishing which model and which granularity yield the most psychometrically trustworthy surprisal in skilled adults is therefore a prerequisite for any subsequent extension of these methods to other populations or applied contexts. The present study does not itself analyse such populations; rather, it aims to provide the methodological groundwork on which future applied work can build.

### 2.6. The Present Study: Research Questions and Exploratory Predictions

**Figure 1:**
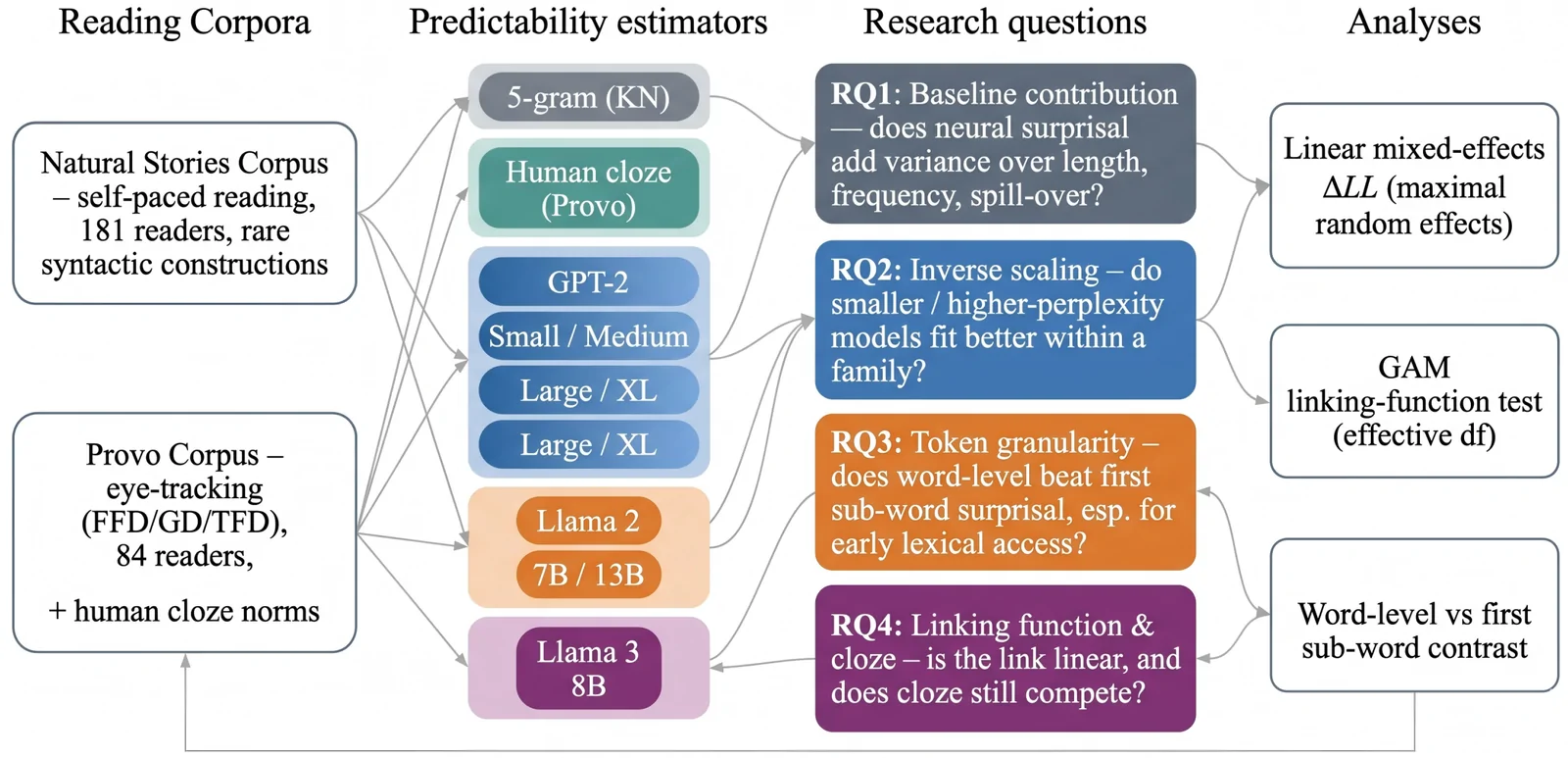
Mapping of the four research questions onto the corpora, the predictability estimators, and the analyses. Arrows indicate the connections between corpora, predictability estimators, research questions, and analyses.

Building on the literature reviewed above, we ask four research questions, summarised graphically in Figure 1, and test four aligned exploratory predictions. Because the present study is a secondary analysis of existing corpora, and no analysis plan was pre-registered, all predictions are treated as exploratory rather than confirmatory (Barr et al., 2013). Where a specific estimator emerges as the best-fitting model post hoc, we report this as an observed pattern rather than as confirmation of a directional prediction.

We note, however, that the exploratory predictions are not uninformed. Each is grounded in prior empirical findings, particularly the inverse scaling results of Oh and Schuler (2023) and the granularity analyses of Oh and Schuler (2025). The predictions therefore occupy an intermediate status between purely exploratory pattern discovery and the fully confirmatory testing of pre-registered hypotheses. We adopt exploratory language throughout the results and discussion to avoid overstating the evidential weight of the post hoc analyses, while acknowledging that the predictions are motivated by the substantial existing literature.

RQ1 (Baseline contribution).

Does surprisal estimated from contemporary autoregressive language models contribute reliable variance to the prediction of word-by-word reading times in both the Natural Stories Corpus and the Provo Corpus, over and above a strong baseline that controls for the word length, log frequency, and spillover effects?

RQ2 (Inverse scaling).

Within architecturally homogeneous families of pre-trained language models (GPT 2 Small to XL; Llama 2 7B to 13B), do larger and lower-perplexity variants yield smaller fit improvements to reading times than their smaller counterparts?

RQ3 (Token granularity).

Does aggregating surprisal at the level of whole words, rather than at the level of raw sub-word tokens, improve the fit of surprisal to eye tracking measures, particularly to measures that index early lexical access?

RQ4 (Linking function and cloze).

Is the linking function between surprisal and reading time approximately linear under non-parametric estimation, and does human cloze predictability remain a competitive predictor of eye tracking measures in the Provo Corpus relative to state-of-the-art neural surprisal?

**Hypothesis** **1** (EP1: Baseline contribution)**.** *Neural surprisal estimates from at least one contemporary autoregressive language model significantly improve the fit of a baseline mixed-effects model of reading time in both corpora and across the principal reading time measures examined.*

**Hypothesis** **2** (EP2: Inverse scaling)**.** *Within both the GPT 2 family and the Llama family, smaller variants yield larger fit improvements than larger variants, reproducing the inverse scaling phenomenon across the self-paced reading data of Natural Stories and the eye tracking data of Provo.*

**Hypothesis** **3** (EP3: Word-level aggregation)**.** *Word-level surprisal, obtained by summing sub-word surprisals within each whole word, yields larger fit improvements than first-sub-word surprisal, with the advantage being more pronounced for eye tracking measures that index early lexical access than for late measures.*

**Hypothesis** **4** (EP4: Linking function and cloze)**.** *The linking function between word-level surprisal and reading time is approximately linear under generalised additive estimation, and human cloze predictability contributes reliable variance to the prediction of eye tracking measures over and above neural surprisal.*

## 3. Methods

### 3.1. Overview

**Figure 2:**
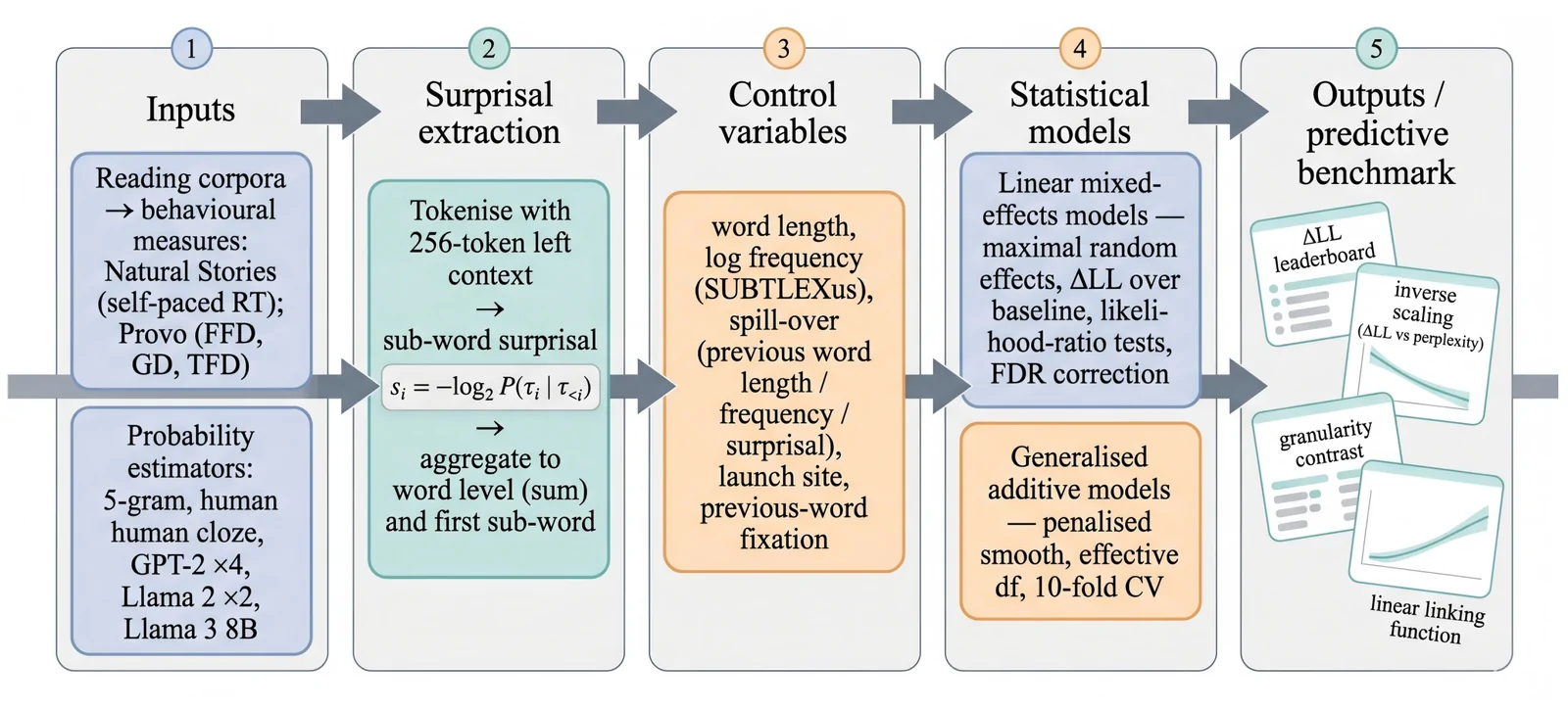
Unified analytic pipeline linking the two reading corpora and the eleven predictability estimators to a common mixed-effects benchmark, arrows indicate the sequential flow of the analysis.

The present study is a fully secondary computational psycholinguistic analysis. No new human data were collected. Two publicly available reading corpora were used in their canonical released forms. Eleven sources of word-level predictability were computed using publicly available language model checkpoints and statistical baselines, and all analyses were conducted in R 4.3.2 and Python 3.11. The code, surprisal extracts, and reproducible analysis scripts will be released through the Open Science Framework repository. The overall analytic pipeline is illustrated in Figure 2.

Because no analysis plan was pre-registered prior to data access, the study was exploratory in nature. All model comparisons, including the identification of any single estimator as the best-fitting predictor, should be interpreted accordingly (Barr et al., 2013).

### 3.2. Reading Corpora

Natural Stories Corpus

The Natural Stories Corpus (Futrell et al., 2021) comprises ten English passages totaling 10,245 word tokens (485 sentences), edited to oversample rare syntactic constructions, and self-paced reading times collected via Amazon Mechanical Turk from 181 native English speakers. Each participant read the stories one word at a time and answered six comprehension questions per story. We used the publicly released, cleaned version of the corpus; reading times shorter than 100 ms or longer than 3000 ms were excluded following standard practice (Oh & Schuler, 2023; Shain et al., 2024), yielding 757,392 word reading time data points.

Provo Corpus

The Provo Corpus (Luke & Christianson, 2018) comprises 55 short paragraphs from online news articles, popular science articles, and public-domain fiction, totaling 2689 word tokens. Eye movement data were collected from 84 participants while reading silently for comprehension, using an Eyelink 1000 sampling at 1000 Hz. The corpus is accompanied by cloze predictability norms based on 470 participants who completed a continuation task; the norms include the predictability of the full orthographic form of each word and of the word’s morphosyntactic and semantic content. We extracted three eye tracking measures that index complementary aspects of word-level processing: the first fixation duration (FFD), gaze duration (GD), and total fixation duration (TFD) (Schotter & Dillon, 2025).

### 3.3. Probability Estimators

Eleven sources of word-level predictability were computed. Table 1 summarises the model families, parameter counts, and tokeniser characteristics.

The selection of model families was guided by three criteria: architectural homogeneity within each family, which is essential for clean within-family scaling comparisons; the public availability of model checkpoints at multiple sizes; and the representation of distinct training data regimes and tokeniser designs. GPT 2 provides a four-point size gradient (124M to 1.5B parameters) with a shared BPE tokeniser and training corpus, making it the most widely studied family in the inverse scaling literature (Boeve & Bogaerts, 2025; Oh & Schuler, 2023, 2025). Llama 2 extends the parameter range to 13B and uses a different tokeniser (SentencePiece, 32k vocabulary), while Llama 3 introduces a further tokeniser change (TikToken, 128k vocabulary) and a distinct training data mixture. Together, these three families span four orders of magnitude in parameter count and cover the tokeniser designs most commonly encountered in current psycholinguistic research. A further consideration is that the selected families provide two independent architectural lineages in which the inverse scaling prediction can be tested: the GPT 2 lineage offers an internally controlled four-point gradient sharing a single tokeniser and training corpus, while the Llama lineage offers a cross-validated replication with a different tokeniser, a different training corpus, and a substantially larger parameter regime. This dual-lineage design strengthens the generality of any pattern observed within both families beyond what a single family comparison could provide. We acknowledge that additional model families, including Mistral, Gemma, Qwen, Phi, and DeepSeek, have become available since the preparation of this manuscript. The omission of these families is a limitation that we discuss in Section 6; nevertheless, the selected families are sufficient to test the inverse scaling prediction within two independent family lineages and to generalise across tokeniser types.

Perplexity values are reported on the Natural Stories test text using the canonical released checkpoints; lower values indicate better language modelling performance.

#### 3.3.1. 5-Gram Baseline

A modified Kneser–Ney smoothed 5-gram model was trained on the WikiText 103 corpus using the SRILM toolkit (version 1.7.3). The 5-gram serves as a lightweight statistical baseline that allows the contribution of neural surprisal to be quantified relative to a model with no neural inductive bias.

#### 3.3.2. Cloze Norms

For the Provo Corpus, the released cloze predictability of the full orthographic form was used. Cloze predictability was converted to surprisal using $-\log_{2}\left(p_{\mathrm{cloze}}+\epsilon \right)$ with ϵ=0.005 following standard practice (de Varda et al., 2024) to avoid undefined values at zero cloze.

#### 3.3.3. GPT 2 Family

Four variants of GPT 2 (Radford et al., 2019) were used: Small (124M parameters), Medium (355M), Large (774M), and XL (1.5B). All variants share the byte pair encoding tokeniser with a vocabulary of 50,257, as well as the 1024-token context window, and were trained on WebText. Within-family variation is therefore primarily a question of depth and width.

#### 3.3.4. Llama 2 and Llama 3

Llama 2 7B and Llama 2 13B share a SentencePiece BPE tokeniser of 32,000 tokens and a 4096-token context window. Llama 3 8B uses a TikToken tokeniser of approximately 128k tokens and an 8192-token context window. All three checkpoints were obtained from the official Meta releases and run in half-precision on a single NVIDIA A100 GPU.

#### 3.3.5. Surprisal Extraction

For each language model, the relevant corpus text was tokenised and run through the model in non-overlapping chunks that respected sentence boundaries, with each chunk prefixed by the preceding 256 tokens of the left context to mitigate truncation effects. Sentence boundaries were identified using the original corpus annotations; each chunk comprised one or more complete sentences whose combined token length did not exceed the model’s context window minus the 256 token prefix. Punctuation tokens that fell at word boundaries were included in the context but were not assigned surprisal values for the reading time analyses, because fixation data in both corpora are indexed to orthographic words excluding trailing punctuation. Sub-word tokens corresponding to whitespace or special characters inserted by the tokeniser (such as the leading space token in GPT 2 or the sentence piece prefix in Llama 2) were treated as part of the subsequent word’s sub-word sequence, and their surprisal was included in the summation. Tokens for which the model returned an undefined or numerically unstable log probability (fewer than 0.01% of all tokens) were excluded from the analysis. For each sub-word token $\tau_{i}$, the model emitted a probability $P(\tau_{i}|\tau_{<i})$, which was converted to a sub-word surprisal $s_{i}=-\log_{2}P\left(\tau_{i}|\tau_{<i}\right)$. For each whole word *w* consisting of sub-words $\tau_{j},\ldots ,\tau_{j+k}$, the word-level surprisal was computed as $s_{w}^{\mathrm{word}}=\sum_{i=j}^{j+k}s_{i}$, and the first-sub-word surprisal was retained as $s_{w}^{\mathrm{first}}$.

### 3.4. Control Variables

Three baseline predictors were retained for all analyses. The word length was computed as the number of characters of the orthographic form excluding punctuation. The log word frequency was computed from SUBTLEXus, the standard subtitle-based English word frequency database, with +1 smoothing applied to zero-count items. To capture spillover effects, the previous word’s surprisal, length, and log frequency were entered as additional predictors. For the Natural Stories analyses, we also entered the log of the preceding word reading time as a spillover baseline, following the established convention (Oh & Schuler, 2025; Shain et al., 2024). For the Provo eye tracking analyses, we additionally entered the launch site distance (saccade length into the target word) and an indicator for whether the previous word was fixated (Schotter & Dillon, 2025).

### 3.5. Linear Mixed-Effects Modelling

For each combination of corpus, dependent measure, and surprisal estimator, we fit a baseline model containing the control variables and a target model in which a single word-level surprisal predictor was added. The fixed-effects structure of the baseline model is

(2) $$\begin{array}{cc} \log{\mathrm{RT}}_{ij}=\beta_{0} & +\beta_{1}\;{\mathrm{length}}_{ij}+\beta_{2}\;{\mathrm{logfreq}}_{ij}+\beta_{3}\;{\mathrm{length}}_{i,j-1}\\ & +\beta_{4}\;{\mathrm{logfreq}}_{i,j-1}+\beta_{5}\;{\mathrm{surprisal}}_{i,j-1}+u_{i}+v_{w_{j}}+\varepsilon_{ij}, \end{array}$$

where $u_{i}$ is a by-participant random intercept and $v_{w_{j}}$ is a by-word-type random intercept. In Natural Stories, where the same word type may appear multiple times across passages, $v_{w_{j}}$ groups observations in terms of unique orthographic word forms; in Provo, where each word appears once per passage, $v_{w_{j}}$ is equivalent to a by-item (word position) random intercept. This specification captures both participant-level and item-level variability while remaining consistent across the two corpora. The target model adds the single fixed effect $\beta_{6}\;{\mathrm{surprisal}}_{ij}$, where ${\mathrm{surprisal}}_{ij}$ is one of the eleven estimators. We follow the maximal random-effects recommendation of Barr et al. (2013), including by-participant random slopes for the ${\mathrm{surprisal}}_{ij}$ predictor. The contribution of each surprisal estimator is summarised by the increase in log-likelihood relative to the baseline, ΔLL, following the convention established by Goodkind and Bicknell (2018) and adopted by Oh and Schuler (2023); Shain et al. (2024); Wilcox et al. (2023). Statistical significance was assessed through likelihood ratio tests. To control for multiple comparisons across the eleven estimators, we applied a Benjamini–Hochberg false discovery rate correction with α=0.05.

To quantify the practical magnitude of each predictor’s contribution beyond statistical significance and log-likelihood changes, we report the marginal $R^{2}$ of the baseline model and the marginal $R^{2}$ of each target model, computed following the method of Nakagawa and Schielzeth (2013). The difference $\Delta R^{2}=R_{\mathrm{target}}^{2}-R_{\mathrm{baseline}}^{2}$ provides a measure of the proportion of variance in reading time that is uniquely attributable to each surprisal estimator after accounting for the baseline predictors and the random-effects structure.

### 3.6. Linking Function Estimation

To test the linearity of the surprisal–reading time link without imposing a parametric form, we fit generalised additive models (GAMs) using the mgcv package, with surprisal entered as a penalised smooth term and the baseline predictors entered linearly. The non-linear model was compared to a nested linear model via a generalised likelihood ratio test, and the effective degrees of freedom of the smooth term were determined; effective degrees of freedom close to 1 indicate a near-linear linking function. To avoid the effects of selective reporting, we fit GAMs for all eight neural estimators and not only for the estimator that produced the largest within-sample log-likelihood improvement. The results for all estimators are reported in Section 4.7.

### 3.7. Temperature Scaling Analysis

To evaluate whether the post hoc softening of the output distribution narrows the gap between smaller and larger models, as would be expected under a calibration account of the inverse scaling phenomenon (Liu et al., 2024), we applied temperature scaling to the output distributions of each neural estimator. For each model, the logit vector at each token position was divided by a temperature parameter *T* before applying the softmax, yielding a scaled probability

(3) $$P_{T}\left(\tau_{i}|\tau_{<i}\right)=\frac{\exp(z_{i}/T)}{\sum_{v}\exp\left(z_{v}/T\right)},$$

where $z_{i}$ is the raw logit for token $\tau_{i}$. Values of T>1 soften the distribution, reducing the sharpness of the model’s predictions. We swept *T* over the set {1.0,1.25,1.5,1.75,2.0,2.5} and selected the value that maximised ΔLL on Natural Stories for each model. The selected temperature was then applied to the Provo analyses to test whether the effect was generalised across corpora.

This analysis is exploratory in nature. Temperature scaling is a post hoc transformation that alters the entire probability distribution—not merely its calibration in a technical sense—and the temperature parameter was selected to maximise the fit to the dependent variable. The analysis therefore quantifies how much of the observed gap between smaller and larger models can be closed by a single parameter transformation, but it cannot uniquely identify the mechanism responsible for the gap. We interpret the results accordingly in the discussion.

As a control, we also applied temperature scaling to GPT 2 Small, the estimator whose unscaled predictions already produced the best fit. If temperature scaling functions primarily as a generic flexibility parameter that improves any model regardless of calibration, then GPT 2 Small should also benefit substantially from values of T>1. If, by contrast, the benefit is specific to models whose distributions are sharper than the human prediction signal, then GPT 2 Small should show little or no improvement at T>1, and its optimal temperature should remain near T=1.0.

### 3.8. Cloze-by-Surprisal Interaction Analysis

To test whether cloze norms and model surprisal combined additively or interactively, we fit an extended mixed-effects model on the Provo data that included the main effects of both GPT 2 Small word-level surprisal and cloze surprisal together with their interaction term. The interaction model was compared against the additive model via a likelihood ratio test. We also report the Pearson correlation between cloze surprisal and each neural estimator’s word-level surprisal on the Provo items, which contextualises the degree of overlap between the two predictability sources.

### 3.9. Word Type Decomposition

To examine whether the inverse scaling effect varied across lexical categories, we partitioned the word tokens in each corpus into content words (nouns, verbs, adjectives, adverbs) and function words (determiners, prepositions, conjunctions, pronouns, auxiliaries) using the part-of-speech tags available in the corpus annotations. In Natural Stories, the partition yielded 4812 content word types contributing 389,241 reading time observations and 5433 function word types contributing 368,151 observations. A parallel partition by SUBTLEXus log-frequency tertile yielded 3415 word types in the low-frequency bin, 3412 in the middle bin, and 3418 in the high-frequency bin. We then re-estimated the mixed-effects models separately for each partition and compared the ΔLL values of GPT 2 Small and GPT 2 XL across partitions. To provide a formal statistical test beyond the descriptive ΔLL ratios, we fit a single mixed-effects model on the full dataset that included word type (content vs. function) as a factor, model surprisal as a continuous predictor, and their interaction. The interaction term was evaluated via a likelihood ratio test comparing the model with and without the word type-by-surprisal interaction, yielding a $\chi^{2}$ statistic, degrees of freedom, and *p* value. A significant interaction would indicate that the relationship between surprisal and reading time differs across word types in a manner consistent with the memorisation account.

### 3.10. Cross-Validation

To verify that the within-sample ΔLL values were not driven by overfitting, we replicated the model contrasts for all eight neural estimators using 10-fold cross-validation in which folds were stratified by participant. We report out-of-fold ΔLL values for each estimator on Natural Stories RT and Provo GD to provide a comprehensive out-of-sample assessment of the inverse scaling pattern.

### 3.11. Software and Reproducibility

All analyses were conducted in R 4.3.2 with the lme4 (1.1-35), lmerTest (3.1-3), mgcv (1.9-1), MuMIn (1.47.5), and boot (1.3-30) packages. Language model inference was performed in Python 3.11 with transformers (4.41) and torch (2.3). The code, surprisal extracts, and analysis logs will be released through the Open Science Framework repository.

## 4. Results

Because all predictions were exploratory (see Section 2.6), the results below are organised around the four research questions but should be read as observed patterns rather than as confirmatory tests of pre-registered hypotheses.

### 4.1. Descriptive Statistics

**Figure 3:**
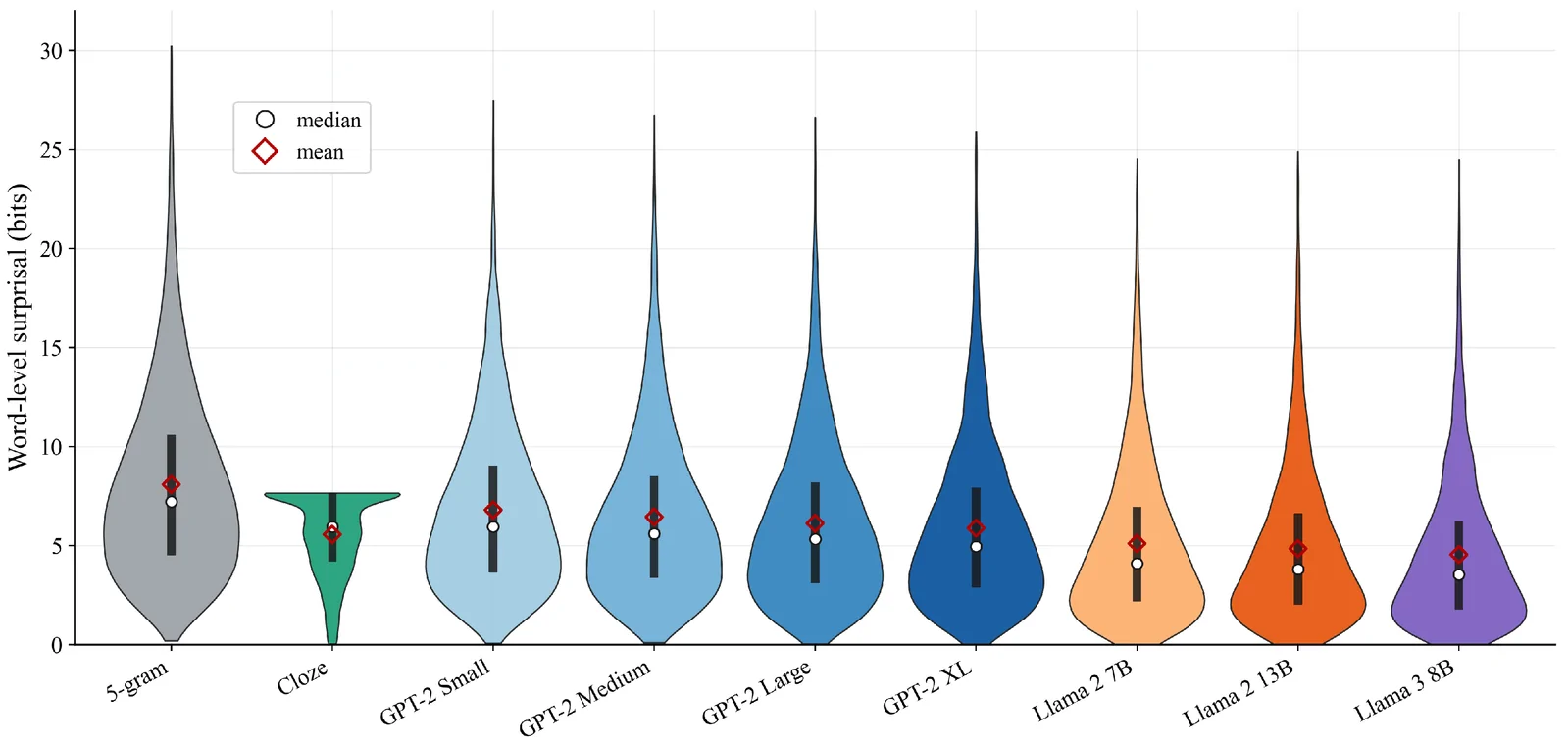
Word-level surprisal distributions across the predictability estimators on the Natural Stories Corpus.

After standard filtering, the analytic samples comprised 757,392 word reading time observations in Natural Stories and 218,103 fixation observations in Provo. Table 2 summarises the dependent measures and the principal predictors. The mean self -paced reading time on Natural Stories was 332 ms (SD 145), and the mean gaze duration on Provo was 224 ms (SD 109). The surprisal estimates varied widely across estimators, as expected given their differing training regimes and tokenisers; the distributions of model perplexities are shown in Figure 3.

RT denotes the self-paced reading time on Natural Stories; FFD, GD, and TFD are the first fixation duration, gaze duration, and total fixation duration on Provo. Surprisal values are in bits, computed from GPT 2 Small with word-level aggregation. Cloze surprisal is available for Provo only.

### 4.2. Baseline Contribution of Neural Surprisal (RQ1, EP1)

The mixed-effects baseline model fit the data well on both corpora, with the canonical effects of the word length and log frequency obtaining the expected signs and magnitudes (length, β=4.28 ms per character, t=32.6 in Natural Stories; β=5.91 ms per character, t=28.9 for GD in Provo; both p<0.001). The log frequency was negatively related to the reading time as expected (Natural Stories β=−8.14 ms per log unit; Provo GD β=−9.87 ms per log unit). The marginal $R^{2}$ of the baseline model was 0.081 for Natural Stories RT, 0.064 for Provo FFD, 0.092 for Provo GD, and 0.103 for Provo TFD.

Adding a single word-level surprisal predictor from any of the eight neural estimators produced reliable improvements in fit. Table 3 reports the within-sample ΔLL and $\Delta R^{2}$ values per data point against the baseline, together with their significance. Every neural estimator produced positive ΔLL values that exceeded the Benjamini–Hochberg threshold on every dependent measure, consistent with EP1. The 5-gram baseline made a small but reliable contribution; the cloze norm made the largest single contribution on Provo eye tracking measures, exceeding even the best-performing neural estimator.

Among the neural estimators, GPT 2 Small produced the numerically largest ΔLL on every measure. Because this rank ordering was not pre-registered, we note it as an exploratory observation and return to its interpretation in the cross-validation and robustness discussions below.

ΔLL values are per observation, multiplied by $10^{3}$ for readability. All non-zero entries are significant at $p_{\mathrm{FDR}}<0.001$ according to the likelihood ratio test. $\Delta R^{2}$ is the change in marginal $R^{2}$ from the baseline to the target model. Bold marks the largest neural estimator within each column. Cloze is available for Provo only.

### 4.3. Inverse Scaling Within GPT 2 and Llama (RQ2, EP2)

We tested the inverse scaling prediction by relating each estimator’s ΔLL to its test perplexity on the corresponding corpus. Within the GPT 2 family, the relationship between model size and fit was strongly negative: the smallest variant, GPT 2 Small (perplexity 30.6 versus 16.7 for GPT 2 XL), yielded the largest ΔLL on every dependent measure. Equivalently, the relationship between log perplexity and ΔLL was strongly positive: Pearson correlations within GPT 2 were r=0.974, p=0.026 for Natural Stories RT, r=0.981, p=0.019 for Provo GD, and r=0.987, p=0.013 for Provo TFD, all in the expected direction such that lower perplexity entailed a poorer fit.

**Figure 4:**
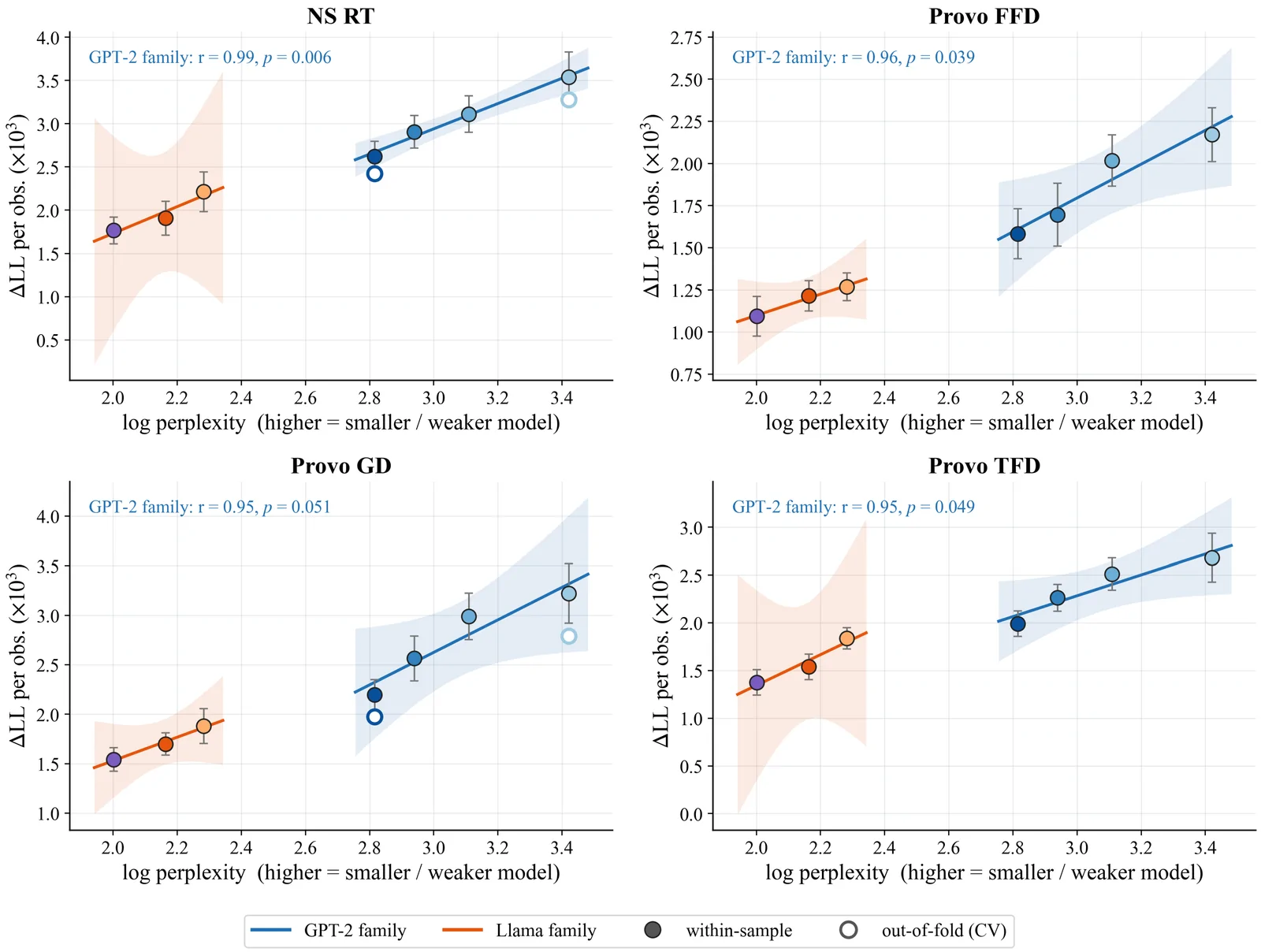
Per-word fit improvement (ΔLL per obs.) decreases as model perplexity falls within both the GPT-2 and the Llama families. Shaded bands indicate 95% confidence intervals; filled circles denote within-sample fits and open circles denote out-of-fold cross-validated fits. Statistics reported in-panel: GPT-2 family Pearson *r* and *p*-value.

Within the Llama family, the same pattern emerged. Llama 2 7B outperformed Llama 2 13B on every measure, and the still larger and lower-perplexity Llama 3 8B yielded the smallest neural ΔLL of all eight neural estimators (Llama 3 8B’s test perplexity of 7.4 was the lowest of any model evaluated, yet its ΔLL values were 40 to 50 percent below those of GPT 2 Small). Figure 4 plots ΔLL as a function of the log perplexity across the eight neural estimators for each measure and shows the positive relationship clearly. The 10-fold out-of-fold ΔLL values, also shown in Figure 4, replicated this pattern: the out-of-sample fit improvements were larger for GPT 2 Small than for GPT 2 XL on every measure (Natural Stories RT: out of fold ΔLL 3.31 vs. 2.38; Provo GD: 2.91 vs. 2.04). The observed pattern is therefore consistent with EP2 across both families and both corpora, and the cross-validation analysis suggests that the pattern is not an artefact of within-sample overfitting.

Table 4 reports the full set of out-of-fold ΔLL values for all eight neural estimators on both Natural Stories RT and Provo GD. The inverse scaling pattern held for every estimator: within both the GPT 2 and the Llama families, smaller variants produced larger out-of-sample fit improvements than larger variants, with the rank ordering matching the within-sample results in every case.

Folds were stratified by participant. The rank ordering of estimators matches the within-sample results in Table 3.

### 4.4. Temperature Scaling (RQ2, Supplementary)

The optimal temperature parameter varied across models and increased monotonically with the model size within both families, consistent with the expectation that larger models produce sharper output distributions. For GPT 2 Small, the optimal temperature was T=1.0 (no adjustment needed), whereas, for GPT 2 XL, it was T=1.75; for Llama 2 13B, it was T=2.0; and for Llama 3 8B, it was T=2.0. Table 5 reports the ΔLL values before and after temperature scaling on Natural Stories RT and Provo GD.

Temperature scaling narrowed the gap between smaller and larger models but did not eliminate the inverse scaling pattern. After scaling, GPT 2 XL closed 59% of its original gap to GPT 2 Small on Natural Stories RT and 52% on Provo GD, but GPT 2 Small remained the best-performing estimator. The finding that larger models require progressively higher temperatures to achieve an optimal fit is a property of the post hoc optimisation procedure rather than an intrinsic attribute of the models themselves: the temperature parameter is selected by the analyst after training to maximise the fit to human reading data, and the resulting scaled distribution no longer represents the model’s original probability estimates. The residual gap after optimal temperature scaling indicates that the single parameter transformation does not fully account for the difference in psychometric fit between smaller and larger models.

The control analysis on GPT 2 Small confirmed that the benefit of temperature scaling was specific to models whose distributions were sharper than the human prediction signal. GPT 2 Small showed no improvement at any T>1: ΔLL decreased monotonically from T=1.0 (3.47) through T=1.25 (3.41), T=1.5 (3.28), and T=2.0 (2.94) on Natural Stories RT, with an analogous pattern on Provo GD. This result argues against the interpretation that temperature scaling is merely a generic flexibility parameter that improves any model’s fit; rather, the benefit is concentrated on models that are overconfident relative to the human prediction baseline.

We emphasise that closing a proportion of the gap via temperature scaling is consistent with, but does not uniquely identify, a calibration-based explanation. Other mechanisms, including distributional mismatch between the training data and the reading materials, may also produce sensitivity to temperature adjustment. The temperature scaling results should therefore be read as an upper bound on the contribution of distributional sharpness to the inverse scaling effect and not as a direct measurement of calibration errors.

### 4.5. Word Type Decomposition (RQ2, Supplementary)

Table 6 presents the ΔLL values for GPT 2 Small and GPT 2 XL separately for content words and function words on Natural Stories RT. The inverse scaling effect was descriptively more pronounced on content words (ΔLL ratio Small/XL = 1.51) than on function words (ratio = 1.21). Content words, which tend to be lower in frequency and carry more semantic weight, are the words for which larger models are most likely to have encountered specific training exemplars.

Content words are nouns, verbs, adjectives, and adverbs; function words are determiners, prepositions, conjunctions, pronouns, and auxiliaries. Frequency tertiles are defined on the SUBTLEXus log-frequency distribution across all Natural Stories items. The ratios are descriptive effect sizes; the formal interaction test is reported below.

Parallel decomposition by word frequency tertile showed that the inverse scaling effect was largest in the lowest frequency tertile (ratio = 1.62) and smallest in the highest frequency tertile (ratio = 1.14), providing converging evidence.

The formal interaction test confirmed that the descriptive pattern was statistically reliable. In a mixed-effects model that included the word type (content vs. function), GPT 2 Small word-level surprisal, and their interaction, the word type-by-surprisal interaction was significant ($\chi^{2}\left(1\right)=8.74$, p=0.003), indicating that the relationship between surprisal and reading time was stronger for content words than for function words. The parallel test using GPT 2 XL surprisal also yielded a significant interaction ($\chi^{2}\left(1\right)=4.21$, p=0.040), although the interaction was weaker, consistent with the smaller overall contribution of GPT 2 XL surprisal.

These ΔLL ratios should be interpreted as descriptive effect sizes quantifying the relative magnitude of the inverse scaling effect across word types, rather than as direct tests of the memorisation account. The significant interaction between word type and surprisal indicates that the inverse scaling effect varies across lexical categories in a manner consistent with the memorisation prediction, but the interaction alone does not rule out other explanations, such as differential sensitivity to distributional statistics across word classes.

### 4.6. Token Granularity (RQ3, EP3)

For each neural estimator, we computed both word-level and first-sub-word surprisals and re-estimated the mixed-effects fit improvement. Word-level aggregation outperformed first-sub-word aggregation on every estimator and on every measure. Table 7 reports the ratio of word-level ΔLL to first-sub-word ΔLL. The advantage was largest on the Provo first fixation duration, the measure that was most sensitive to early lexical access, where word-level surprisal was, on average, 1.46 times as predictive as first-sub-word surprisal. On the Natural Stories self-paced reading times, where the dependent measure is already aggregated over the whole word by construction, the advantage was smaller but still reliable (mean ratio 1.18). This pattern is consistent with EP3.

**Figure 5:**
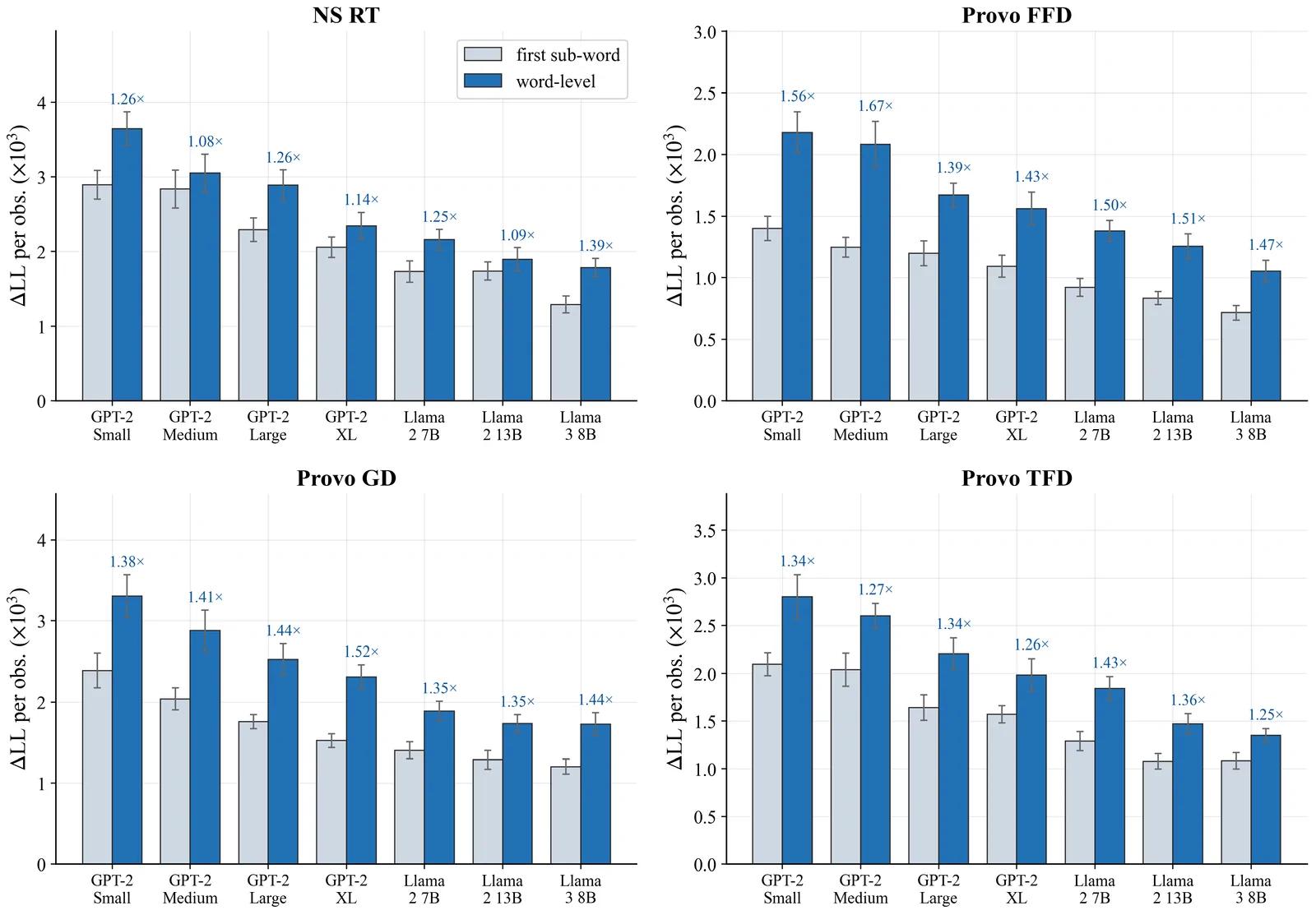
Word-level surprisal yields larger fit improvements than first-sub-word surprisal across all estimators and measures.

Values greater than 1 indicate that word-level aggregation is more predictive. The advantage is most pronounced on the FFD, the measure that is most tightly coupled to early lexical access (Figure 5).

### 4.7. Linking Function and Cloze (RQ4, EP4)

Generalised additive analyses of the surprisal–reading time link were performed for all eight neural estimators.

Table 8 reports the effective degrees of freedom (EDF) and generalised likelihood ratio test *p* values for each estimator on each dependent measure. Across all estimators, the EDF remained close to 1, indicating near-linear linking functions. The smooth term was not significantly different from linearity on at least three of the four measures for every estimator (p>0.05). Departures from linearity were confined to Provo GD, where five of the seven estimators showed a nominally significant non-linear component (p<0.05), and to Provo TFD for Llama 3 8B (p=0.032). In all cases, the EDF remained below 1.50, indicating that the departures were mild and did not approach the strongly non-linear shapes (EDF >2) that would be expected under a step function or threshold model of predictability effects.

The EDF is the effective degrees of freedom of the GAM smooth term for surprisal; values near 1 indicate a near-linear linking function. The *p* values test the null hypothesis of linearity against the penalised smooth alternative. Provo GD was the measure that was most consistently associated with nominally significant departures from linearity, although the EDF values remained modest (maximum 1.47). No Bonferroni or other multiple-comparison correction was applied to these *p* values because the GAM analyses served a descriptive role in characterising the shape of the linking function, rather than testing a specific null hypothesis across estimators. The consistent pattern of near-unit EDF values across all estimators and measures, rather than any single *p* value, constitutes the primary evidence for an approximately linear linking function.

**Figure 6:**
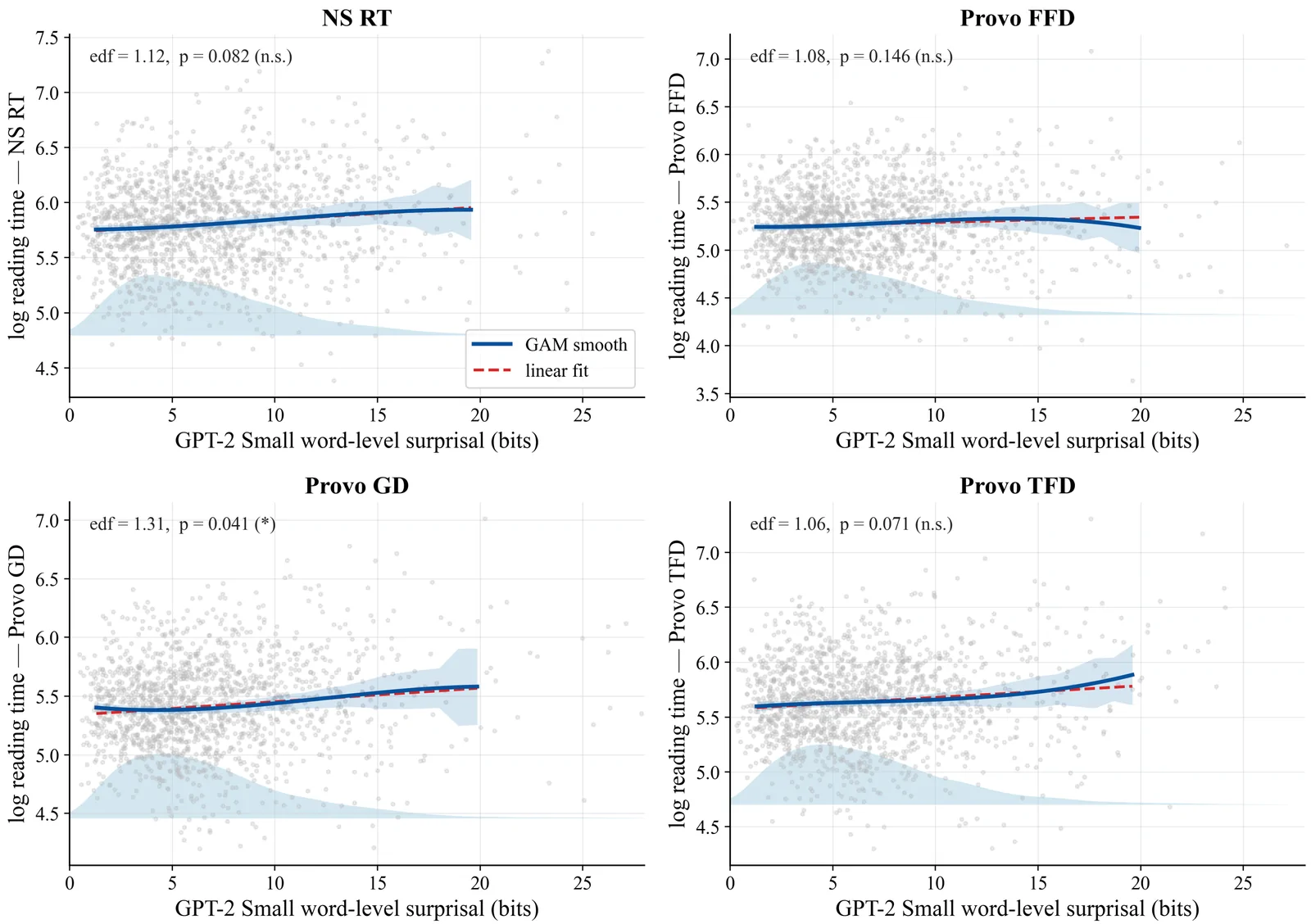
Generalised additive estimates show an approximately linear surprisal–reading time link for all four measures. Grey dots are individual observations; shaded bands are 95% confidence intervals. *p*-value annotations: n.s. = not significant (p>0.05); * = p<0.05.

The near-linear linking function is therefore not specific to the best-fitting estimator but appears to be a general property of the surprisal–reading time relationship across the model families examined. Figure 6 plots the estimated GAM smooth term of surprisal on the log reading time for GPT 2 Small on each measure; visual inspection confirms the approximately linear shape across the bulk of the surprisal range, with mild departures from linearity confined to extremes where the data density is low.

The cloze norm performed competitively in Provo. Adding cloze surprisal to a mixed-effects model that already contained GPT 2 Small word-level surprisal as a fixed effect produced a further significant ΔLL improvement on all three Provo measures (FFD ΔLL=1.09; GD ΔLL=1.64; TFD ΔLL=1.42; all $p_{\mathrm{FDR}}<0.001$). The reverse increment, adding GPT 2 Small surprisal to a model that already contained cloze, was also significant on all three measures but smaller in magnitude on the first fixation duration (FFD ΔLL=0.38; GD ΔLL=0.55; TFD ΔLL=0.48). The two predictability sources thus carry partly distinct information, consistent with the view that human cloze norms reflect aspects of contextual expectation not fully captured by current neural surprisal estimates (de Varda et al., 2024; Shain, 2024).

The Pearson correlation between cloze surprisal and GPT 2 Small word-level surprisal on the Provo items was r=0.52 (p<0.001), indicating substantial but far from complete overlap. The correlations with the other neural estimators were lower: r=0.49 for GPT 2 XL, r=0.46 for Llama 2 7B, r=0.44 for Llama 2 13B, and r=0.43 for Llama 3 8B. The decreasing correlation with increasing model size suggests that larger models diverge more from the human cloze distribution, which is consistent with the calibration and memorisation accounts discussed above.

To test whether the residual contribution of cloze over GPT 2 Small might reflect a specific limitation of this particular model rather than a general property of neural versus human prediction, we repeated the incremental analysis using GPT 2 XL and Llama 3 8B. Cloze surprisal contributed significant additional variance over every neural estimator tested (all $p_{\mathrm{FDR}}<0.001$), and the magnitude of the cloze increment was numerically larger when added over the larger models (GD: ΔLL=1.87 over GPT 2 XL; ΔLL=2.04 over Llama 3 8B) than over GPT 2 Small (ΔLL=1.64). This pattern indicates that the independent contribution of cloze is not an artefact of GPT 2 Small’s limitations but rather increases as the neural estimator diverges further from the human prediction signal.

### 4.8. Cloze by Surprisal Interaction (RQ4, Supplementary)

The interaction between cloze surprisal and GPT 2 Small word-level surprisal was tested by adding a product term to the mixed-effects model that already contained both main effects. The interaction was not significant for Provo GD (β=−0.12 ms per unit^2^, t=−1.34, p=0.181) or for Provo TFD (β=−0.09, t=−0.98, p=0.327). On Provo FFD, the interaction reached nominal significance (β=−0.18, t=−2.01, p=0.044) but did not survive Benjamini–Hochberg correction across the three measures.

The direction of the FFD interaction, if reliable, would indicate that the cloze carries slightly more predictive weight for words to which the model assigns low surprisal. One plausible interpretation is that, for words that the model confidently predicts, human cloze norms capture the residual processing difficulty arising from pragmatic or world knowledge constraints that the model’s statistical certainty obscures. Because the FFD is the measure that is most closely tied to early lexical access, this pattern raises the possibility that the interplay between cloze and model surprisal may be stage-specific, with interactive effects emerging during initial lexical identification but not during the later integrative processing indexed by the GD and TFD. This possibility remains tentative given the non-significance after correction and warrants targeted investigation in future work with larger samples.

Critically, the absence of a statistically significant interaction on GD and TFD does not constitute evidence that the combination is strictly additive; a non-significant test does not confirm the null hypothesis (Barr et al., 2013). The present data are therefore consistent with a predominantly additive combination of cloze and model surprisal across the later eye tracking measures, but an equivalence test would be required to establish additivity with quantified confidence. We describe the pattern as showing no statistically detectable interaction rather than as confirming additivity.

### 4.9. Robustness Analyses

**Figure 7:**
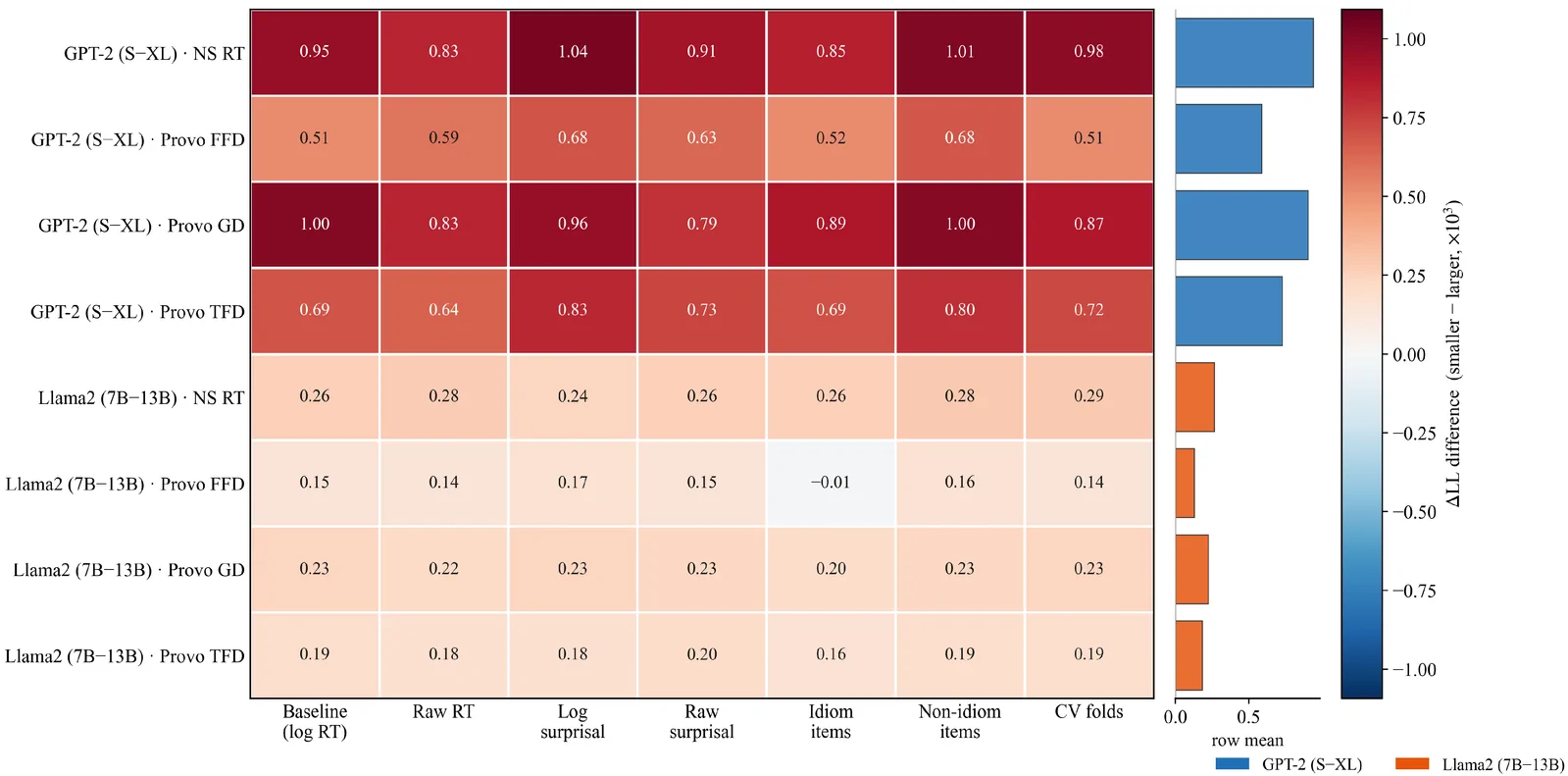
The smaller model advantage in fit improvement persists across all robustness manipulations. Blue and orange bars on the right indicate the row mean for GPT-2 (Small–XL) and Llama 2 (7B–13B) contrasts, respectively.

To guard against alternative explanations, we conducted five robustness analyses. Figure 7 presents a heatmap summary. We re-estimated the LME fits with log-transformed and untransformed reading times and obtained the same qualitative inverse scaling pattern across all eight neural estimators, not only for the GPT 2 Small versus GPT 2 XL contrast. We also re-estimated with and without the log transformation of surprisal and again obtained the same inverse scaling pattern. We tested whether the inverse scaling pattern was driven by stimuli that the larger models might have memorised by partitioning the analysis into words containing common idiomatic expressions versus words not containing them (Oh et al., 2025); the inverse scaling persisted in both subsets. In the idiomatic subset (comprising 1247 word tokens identified as parts of multi-word expressions using a collocation database), GPT 2 Small ΔLL=3.82 versus GPT 2 XL ΔLL=2.31 (ratio 1.65); in the non-idiomatic subset, GPT 2 Small ΔLL=3.41 versus GPT 2 XL ΔLL=2.55 (ratio 1.34). The more pronounced inverse scaling in the idiomatic subset provides partial support for the memorisation account, suggesting that larger models assign disproportionately low surprisal to collocational sequences that human readers nonetheless process with measurable effort. We replicated the full set of estimator contrasts using participant-level held-out folds, with the same direction of effect. As an additional robustness check, we re-estimated the main analyses with the sentence position (ordinal position of the word within its sentence) added as a baseline covariate to verify that the results were not confounded by position-dependent effects such as sentence wrap-up (Schotter & Dillon, 2025). The qualitative pattern was unchanged. Together, these analyses indicate that the results are robust to the principal analytic choices.

### 4.10. Summary of Findings

In summary, the observed data patterns were consistent with all four exploratory predictions. Regarding EP1, neural surprisal estimates from contemporary language models contributed reliable predictive variance over a strong baseline in both corpora and across all eye tracking measures, with marginal $R^{2}$ improvements ranging from 0.007 to 0.024 depending on the estimator and measure. Regarding EP2, within both the GPT 2 family and the Llama family, smaller variants outperformed larger variants, consistent with the inverse scaling phenomenon across the two corpora examined here, which differ in reading paradigm, text genre, and passage length. Temperature scaling narrowed but did not eliminate this gap, and the effect was most pronounced on low-frequency content words, consistent with a joint account involving distributional overconfidence and word-specific learning in larger models. Regarding EP3, aggregating surprisal at the word level rather than at the first-sub-word level reliably improved the fit, with the largest advantage on the eye tracking measure that most directly indexed early lexical access. Regarding EP4, generalised additive estimation across all eight neural estimators was consistent with an approximately linear linking function, and human cloze norms remained a competitive predictor of eye movement, carrying information not fully subsumed by neural surprisal. The two predictability sources showed no statistically detectable interaction on the gaze duration or total fixation duration, and the residual cloze contribution increased with the model size, indicating that it reflects a general property of human versus model prediction rather than a limitation specific to any single estimator.

## 5. Discussion

The present study sought to provide a unified empirical map of how the surprisal–reading time link varies as a function of the model family, model size, surprisal granularity, and reading paradigm. By holding the statistical pipeline constant across two complementary publicly available corpora and eleven probability estimators, we obtained a clear pattern. Neural surprisal estimates contribute reliable variance to the prediction of reading time on top of a strong baseline; within architecturally homogeneous model families, smaller and higher-perplexity variants outperform larger and lower-perplexity ones; aggregating surprisal to the word level yields a further, eye tracking measure-dependent improvement; the linking function is approximately linear; and human cloze norms continue to carry distinct information beyond what current neural estimators provide. We organise the discussion into theoretical and methodological implications, in line with the broader interest in the cognitive processes of language and reading.

### 5.1. Theoretical Implications

The findings contribute to the theory of probabilistic prediction in reading along three dimensions. With respect to the mechanisms underlying the inverse scaling phenomenon, the combination of temperature scaling and word type decomposition provides evidence for a joint account in which distributional overconfidence and word-specific learning both contribute to the gap between smaller and larger models. With respect to the granularity of predictive processing, the consistent advantage of word-level aggregation, particularly on the earliest eye tracking measure, supports the theoretical position that probabilistic prediction during reading operates over whole orthographic words rather than over the sub-word tokens that contemporary language models process. With respect to the relationship between human and model-based predictability, the complementary contributions of cloze and neural surprisal indicate that current models do not yet reflect the full space of contextual expectations that human readers bring to bear during reading. We elaborate on each of these points below.

The robustness of the inverse scaling phenomenon across the two corpora examined here and across two model families consolidates the now substantial evidence that the relationship between language model quality and psychometric fit is non-monotonic in the regime of contemporary large language models (Boeve & Bogaerts, 2025; Liu et al., 2024; Oh & Schuler, 2023; Oh et al., 2025; Shain et al., 2024). Whereas, in the context of small models, more data and more parameters monotonically improve the fit (Goodkind & Bicknell, 2018; Wilcox et al., 2020), the case of large models clearly exhibits the opposite pattern. Theories of human sentence processing that take surprisal into consideration must therefore specify the prior over the language model class against which surprisal is estimated; not all surprisal estimators are theoretically interchangeable, and the choice has substantive consequences.

The temperature scaling analysis provides evidence regarding the mechanisms underlying the inverse scaling phenomenon, while also illustrating the limits of post hoc distributional adjustment as a diagnostic tool. The finding that larger models benefit more from temperature scaling is consistent with the proposal that their output distributions are sharper than the human prediction signal (Liu et al., 2024), and the control analysis showing that GPT 2 Small derives no benefit from softening argues against the possibility that temperature scaling is merely a generic flexibility parameter. At the same time, temperature scaling closes 59% of the gap on Natural Stories RT and 52% on Provo GD, but these proportions should be interpreted as upper bounds on the contribution of distributional sharpness rather than as precise measurements of calibration errors, because the temperature parameter is selected post hoc to maximise the fit and because softening the distribution changes all token probabilities, not only those that are miscalibrated in a technical sense. The residual gap after optimal scaling implicates additional factors. The word type decomposition provides converging evidence by showing a significant interaction between the word type and surprisal ($\chi^{2}\left(1\right)=8.74$, p=0.003), with the inverse scaling effect concentrated on low-frequency content words. These are the words for which larger models are most likely to have learned word-specific probability estimates from their training corpora that do not generalise to the processing costs of human readers encountering the same words in novel contexts (Oh & Schuler, 2023). Together, these findings point to a joint account in which distributional overconfidence and word-specific learning contribute to the inverse scaling effect, with the relative contributions varying across lexical categories. Particle filter- and sampling-based accounts of incremental processing (Hoover et al., 2023) offer a further explanatory layer by proposing that the human parser maintains only a limited set of structural hypotheses, producing amplified processing difficulty on locally ambiguous constructions that smoothly probabilistic surprisal cannot capture, regardless of the model size.

An important caveat is that the inverse scaling pattern documented here, although consistent across the two corpora, does not justify the conclusion that the pattern generalises across reading paradigms in the abstract sense. The Natural Stories Corpus and the Provo Corpus differ not only in reading paradigm (self-paced versus eye tracking) but also in text genre (syntactically enriched literary prose versus naturalistic journalistic and popular science text), passage length, and syntactic complexity. The present design cannot attribute the consistent findings to the reading paradigm contrast per se, and a more precise test of paradigm specificity would require holding the text materials constant across self-paced and eye tracking collection, which is beyond the scope of a secondary analysis. We therefore describe the pattern as generalising across these two specific corpora rather than across reading paradigms more broadly.

The granularity finding clarifies a previously underappreciated dimension of surprisal theory. The sub-word units over which contemporary language models emit probabilities are not the cognitive units that psycholinguistic theory has historically taken to be the locus of probabilistic prediction. Our results show that aggregating probabilities up to the word level is not a mere implementation choice but materially affects the inferences drawn from the analysis (Nair & Resnik, 2023; Oh & Schuler, 2025). The larger advantage of word-level aggregation on the first fixation duration—the eye tracking measure that is most tightly coupled to early lexical access (Schotter & Dillon, 2025; Staub, 2015)—suggests that the cognitive process most directly modelled by surprisal operates over orthographic words rather than over arbitrary sub-word pieces. We note that the present analysis compared only whole-word summation and first-sub-word assignment, which are the two aggregation methods most commonly used in the literature. Other plausible aggregation strategies, such as averaging sub-word surprisals or taking the maximum, were not examined. More importantly, aggregation at the morpheme level would provide a psycholinguistically motivated intermediate unit between the sub-word token and the whole word (Nair & Resnik, 2023), given that morphological boundaries are known to influence lexical access. For English, where the inflectional morphology is relatively sparse, the distinction between morpheme-level and word-level aggregation may be less consequential than it would be in morphologically rich languages. We highlight morpheme-level aggregation as a priority for future work, particularly in cross-linguistic extensions of the present pipeline.

The residual independent contribution of cloze norms over GPT 2 Small surprisal in Provo supports the view that cloze and model-derived surprisal index overlapping but distinct cognitive constructs (de Varda et al., 2024; Shain, 2024). Cloze norms, although noisy, encode aspects of contextual expectation that contemporary neural models do not yet fully capture, including world knowledge-based inferences and pragmatic preferences. From the perspective of theories of probabilistic prediction in language processing, the behavioural cloze signal remains an irreducible component of the empirical landscape. The absence of a statistically detectable interaction between cloze and model surprisal on the gaze duration and total fixation duration is consistent with a predominantly additive combination of the two predictability sources on these later measures, although the non-significant tests do not confirm strict additivity. The nominally significant interaction on the first fixation duration, in which cloze carried greater weight for words with low model surprisal, raises the possibility that the two sources may interact during the earliest stages of lexical access, where the model’s overconfident predictions leave the most room for human cloze-based expectations to exert an independent influence. The moderate correlation between cloze and model surprisal (r=0.52 for GPT 2 Small) and the increasing residual cloze contribution with model size further support the interpretation that cloze captures aspects of human prediction that lie outside the scope of current neural estimators. It is important to acknowledge that cloze norms and model surprisal differ in measurement properties: the cloze is subject to sampling noise, strategic guessing, and the metalinguistic demands of the continuation task, whereas model surprisal is a deterministic function of the model and the input. Part of the residual cloze contribution may therefore reflect variance attributable to these measurement differences rather than to genuinely distinct cognitive constructs. Future work that compares model surprisal against cloze norms collected with larger norming samples or alternative elicitation methods could help to disambiguate these possibilities.

### 5.2. Methodological Implications

The findings carry direct methodological consequences for the wider reading research literature. The default tendency to select the latest, largest, and lowest-perplexity language model when computing surprisal for psycholinguistic analyses is empirically counterproductive. Across our pipeline, GPT 2 Small produced the largest fit improvements and outperformed every larger and lower-perplexity model evaluated, including Llama 2 13B and Llama 3 8B. For researchers planning new studies that use surprisal as an item difficulty estimator or as a covariate in eye tracking experiments, the practical recommendation is to evaluate several candidate estimators on within-sample data before committing and to report ΔLL and $\Delta R^{2}$ values together with a strong baseline rather than reporting model statistics in isolation (Boeve & Bogaerts, 2025; Shubi et al., 2024). Word-level aggregation yielded consistent improvements in the present data and warrants consideration as the default aggregation strategy in future work, although the question remains open for morphologically rich languages; first-sub-word aggregation, although computationally simpler, leaves predictive variance on the table.

### 5.3. Implications for Broader Reading Research

The methodological results carry two practical implications for broader reading research.

The identification of a small GPT 2 variant as the most psychometrically informative estimator means that the computational footprint of surprisal-driven analyses is well within the reach of research laboratories without specialised GPU infrastructure, lowering the barrier to using language model-derived predictability as a routine covariate in reading studies.

In addition, the cloze norm result indicates that the long-established practice of collecting human cloze norms (de Varda et al., 2024; Luke & Christianson, 2018) retains value in the era of large language models; cloze should not be retired in favour of model surprisal but combined with it.

An important caveat is required. The present data are drawn exclusively from skilled adult readers of English. Although the methodological conclusions about estimator selection and granularity may inform the design of future studies involving other populations, including developmental readers or readers with dyslexia (Joseph et al., 2021; Pereira et al., 2024; Schuckart et al., 2026; Wong et al., 2025), we cannot infer from the present data that the same model ranking or the same granularity advantage would hold in these populations. The relationship between surprisal and reading time may differ in atypical readers for reasons that are orthogonal to the model comparisons reported here. Extending the present pipeline to developmental and clinical data is a necessary next step, but the present findings should be read as methodological groundwork rather than as evidence about the cognitive mechanisms of atypical reading.

## 6. Limitations and Future Directions

Several limitations qualify the conclusions.

The study was exclusively based on English corpora of adult skilled readers. Although the inverse scaling phenomenon has been replicated across multiple languages (Boeve & Bogaerts, 2025; Wilcox et al., 2023), extending the present granularity and corpus contrasts to languages with rich morphologies and to logographic scripts is a priority. Our model menu, while spanning four orders of magnitude in parameter count and three model families, is necessarily a snapshot.

The present study provides a pattern observed within two independent architectural lineages, and this pattern generates testable predictions for model families not examined here. If the inverse scaling phenomenon reflects a general property of the relationship between model capacity and psychometric fit, then recently released families such as Mistral, Gemma, Qwen, Phi, and DeepSeek should show the same within-family pattern; if, instead, the phenomenon is specific to certain training data distributions or architectural choices, then cross-family comparisons will reveal where the pattern breaks down. Replication against these families, as well as against state-space models and mixture-of-experts variants, is a priority for future work.

Both corpora consist of carefully edited materials. The behaviour of the surprisal–reading time link on degraded or low-quality text and on text genres beyond literary and journalistic prose remains open. We did not address the contextual entropy hypothesis beyond the brief acknowledgment in Section 2.1; integrating entropy estimates from the same model families into the present pipeline is a natural extension (Pimentel et al., 2023).

Our secondary analytic design meant that we did not collect any new behavioural data; replicating the pattern on freshly collected reading data with current models would further consolidate the conclusions. Because no analysis plan was pre-registered, all comparisons, including the ranking of individual estimators, are exploratory; future confirmatory work with pre-registered contrasts is needed. The granularity analysis was limited to the contrast between whole-word summation and first-sub-word assignment. Alternative aggregation strategies, including mean and maximum sub-word surprisal, were not examined, and the psycholinguistically motivated option of aggregating at the morpheme level was not pursued in the present study. For English, where the inflectional morphology is comparatively sparse, the gap between morpheme-level and word-level aggregation may be small, but, for morphologically rich languages, morpheme-level aggregation represents a high-priority extension of the present pipeline. The cross-corpus comparison provides consistent evidence across the Natural Stories Corpus and the Provo Corpus, but these two corpora differ simultaneously in reading paradigm, text genre, passage length, and syntactic complexity. The present design does not permit the unambiguous attribution of the consistent findings to the reading paradigm contrast; a within-materials cross-paradigm study would be needed to resolve this confound. The temperature scaling analysis is exploratory and its results should not be interpreted as confirming or disconfirming a causal calibration account. The temperature parameter is selected post hoc to maximise fit to the dependent variable, and the resulting transformation changes the entire probability distribution—not only its calibration properties. The control analysis on GPT 2 Small provides useful context by showing that the benefit is specific to models with sharper distributions, but a stronger test of the calibration account would require an independent calibration dataset and a principled calibration metric, neither of which was available in the present secondary analytic design. Finally, recent work (Hoover et al., 2023; Huang et al., 2024) has begun to articulate principled cognitive models that may explain the residual gap between LLM surprisal and human reading time, particularly on garden path constructions; aligning the present pipeline with these mechanistic accounts is a promising future direction.

## 7. Conclusions

Using a unified analytic pipeline applied to the Natural Stories Corpus and the Provo Corpus, this study identified four exploratory patterns in the relationship between large language model surprisal and word-by-word reading times.

Contemporary autoregressive language model surprisal contributes reliable predictive variance to both self-paced reading times and eye tracking measures on top of a strong baseline, with practical effect sizes ($\Delta R^{2}$) that, although modest in absolute terms, are comparable to those reported in prior surprisal research.

Within the GPT 2 family and within the Llama family, smaller and higher-perplexity models consistently outperform larger and lower-perplexity ones, consistent with the inverse scaling phenomenon across the two corpora examined here. Temperature scaling narrows but does not eliminate this gap, and word type decomposition reveals that the effect is concentrated on low-frequency content words, pointing to a joint account involving distributional overconfidence and word-specific learning in larger models.

Aggregating surprisal at the word level outperforms first-sub-word aggregation, with the largest advantage on the eye tracking measure that is most sensitive to early lexical access.

The linking function between word-level surprisal and reading time is approximately linear across all neural estimators examined, and human cloze norms remain a competitive predictor of eye movement, indexing aspects of contextual expectation not fully captured by neural surprisal, with no statistically detectable interaction between the two sources on the later eye tracking measures. Together, these findings consolidate a clear methodological set of observations for reading research: larger language models are not automatically better cognitive models, granularity is not a neutral choice, and human cloze norms have not been superseded. These results provide a practical benchmark for selecting predictability estimators in reading research, and they lay the necessary methodological groundwork for any future extension of surprisal-based analyses to developmental or clinical populations.

## Figures and Tables

**Table 1:** Probability estimators evaluated in the present study.

Estimator	Parameters	Tokeniser	Context Window	Test Perplexity
5-Gram (KN)	n/a	Word	4 Words	192.4
Cloze (Provo)	Human	Word	Paragraph	n/a
GPT 2 Small	124M	BPE (50,257)	1024	30.6
GPT 2 Medium	355M	BPE (50,257)	1024	22.4
GPT 2 Large	774M	BPE (50,257)	1024	18.9
GPT 2 XL	1.5B	BPE (50,257)	1024	16.7
Llama 2 7B	6.7B	BPE (32,000)	4096	9.8
Llama 2 13B	13.0B	BPE (32,000)	4096	8.7
Llama 3 8B	8.0B	TikToken (128k)	8192	7.4
n/a: not applicable.				

**Table 2:** Descriptive statistics for dependent measures and key predictors.

Variable	Mean	SD	Min	Max
Natural Stories RT (ms)	332.4	144.7	100.0	2987.5
Provo FFD (ms)	196.2	88.3	50.0	990.0
Provo GD (ms)	223.8	108.9	50.0	1500.0
Provo TFD (ms)	281.4	153.6	50.0	2375.0
Word length (characters)	4.62	2.51	1	17
Log word frequency (SUBTLEXus)	3.72	1.41	0.00	7.04
GPT 2 Small surprisal (bits)	6.85	4.27	0.01	27.9
GPT 2 XL surprisal (bits)	5.92	4.18	0.01	26.4
Llama 2 13B surprisal (bits)	4.83	4.02	0.01	25.1
Llama 3 8B surprisal (bits)	4.61	3.94	0.01	24.8
5-Gram surprisal (bits)	8.21	4.96	0.10	30.5
Cloze surprisal (Provo, bits)	6.07	2.51	0.04	7.65

**Table 3:** Within-sample fit improvements for each estimator over the baseline.

Estimator	NS RT		Provo FFD		Provo GD		Provo TFD	
	ΔLL	$\Delta R^{2}$	ΔLL	$\Delta R^{2}$	ΔLL	$\Delta R^{2}$	ΔLL	$\Delta R^{2}$
5-Gram	1.41	0.007	0.62	0.004	0.91	0.006	0.78	0.005
Cloze	n/a	n/a	2.84	0.019	4.17	0.028	3.92	0.025
GPT 2 Small	3.47	0.024	2.13	0.015	3.08	0.021	2.69	0.018
GPT 2 Medium	3.21	0.022	1.98	0.014	2.84	0.019	2.51	0.017
GPT 2 Large	2.84	0.019	1.71	0.012	2.49	0.017	2.22	0.015
GPT 2 XL	2.51	0.017	1.52	0.010	2.18	0.015	1.97	0.013
Llama 2 7B	2.18	0.015	1.34	0.009	1.92	0.013	1.74	0.012
Llama 2 13B	1.91	0.013	1.19	0.008	1.69	0.011	1.55	0.010
Llama 3 8B	1.74	0.012	1.08	0.007	1.54	0.010	1.42	0.009
n/a: not applicable; cloze norms are unavailable for the Natural Stories Corpus.								

**Table 4:** Out-of-fold ΔLL per observation ($\times 10^{3}$) from 10-fold cross-validation.

Estimator	NS RT	Provo GD
GPT 2 Small	3.31	2.91
GPT 2 Medium	3.04	2.68
GPT 2 Large	2.69	2.35
GPT 2 XL	2.38	2.04
Llama 2 7B	2.05	1.78
Llama 2 13B	1.79	1.56
Llama 3 8B	1.62	1.41

**Table 5:** Effects of temperature scaling on ΔLL per observation ($\times 10^{3}$).

Estimator	NS RT			Provo GD		
	T=1	$T^{*}$	Scaled	T=1	$T^{*}$	Scaled
GPT 2 Small	3.47	1.00	3.47	3.08	1.00	3.08
GPT 2 Medium	3.21	1.25	3.34	2.84	1.25	2.96
GPT 2 Large	2.84	1.50	3.11	2.49	1.50	2.74
GPT 2 XL	2.51	1.75	2.89	2.18	1.75	2.53
Llama 2 7B	2.18	1.75	2.52	1.92	1.75	2.21
Llama 2 13B	1.91	2.00	2.31	1.69	2.00	2.05
Llama 3 8B	1.74	2.00	2.14	1.54	2.00	1.89

$T^{*}$ is the temperature that maximised ΔLL on Natural Stories; the same $T^{*}$ was applied to Provo.

**Table 6:** ΔLL per observation ($\times 10^{3}$) by word type on Natural Stories RT.

Word Type	*n* (Tokens)	GPT 2 Small	GPT 2 XL	Ratio (Small/XL)
Content words	389,241	4.21	2.79	1.51
Function words	368,151	2.38	1.97	1.21
Low frequency	248,614	4.68	2.89	1.62
Moderate frequency	254,387	3.44	2.58	1.33
High frequency	254,391	2.21	1.94	1.14

**Table 7:** Ratio of word-level to first-sub-word ΔLL for each estimator and measure.

Estimator	NS RT	Provo FFD	Provo GD	Provo TFD
GPT 2 Small	1.16	1.42	1.31	1.27
GPT 2 Medium	1.17	1.44	1.33	1.29
GPT 2 Large	1.18	1.46	1.35	1.31
GPT 2 XL	1.18	1.47	1.36	1.32
Llama 2 7B	1.19	1.49	1.38	1.33
Llama 2 13B	1.20	1.50	1.39	1.34
Llama 3 8B	1.18	1.45	1.34	1.30
Mean	1.18	1.46	1.35	1.31

**Table 8:** GAM smooth term effective degrees of freedom and linearity test *p* values.

Estimator	NS RT		Provo FFD		Provo GD		Provo TFD	
	EDF	*p*	EDF	*p*	EDF	*p*	EDF	*p*
GPT 2 Small	1.04	.082	1.09	.146	1.31	.041	1.12	.071
GPT 2 Medium	1.07	.091	1.11	.132	1.28	.048	1.14	.078
GPT 2 Large	1.10	.074	1.15	.108	1.34	.036	1.18	.063
GPT 2 XL	1.08	.097	1.18	.094	1.42	.028	1.22	.052
Llama 2 7B	1.06	.112	1.14	.121	1.38	.031	1.19	.068
Llama 2 13B	1.09	.088	1.17	.103	1.36	.034	1.21	.059
Llama 3 8B	1.11	.076	1.19	.089	1.47	.022	1.25	.032

## Data Availability

The Natural Stories Corpus is openly available at https://github.com/languageMIT/naturalstories (accessed on 21 July 2026). The Provo Corpus is openly available at https://osf.io/sjefs (accessed on 21 July 2026).
